# Orthognathic Surgery and Relapse: A Systematic Review

**DOI:** 10.3390/bioengineering10091071

**Published:** 2023-09-10

**Authors:** Angelo Michele Inchingolo, Assunta Patano, Fabio Piras, Elisabetta de Ruvo, Laura Ferrante, Angela Di Noia, Leonardo Dongiovanni, Andrea Palermo, Francesco Inchingolo, Alessio Danilo Inchingolo, Gianna Dipalma

**Affiliations:** 1 Department of Interdisciplinary Medicine, University of Bari “Aldo Moro”, 70124 Bari, Italy; angeloinchingolo@gmail.com (A.M.I.); assuntapatano@gmail.com (A.P.); dott.fabio.piras@gmail.com (F.P.); studio.deruvo@libero.it (E.d.R.); lauraferrante79@virgilio.it (L.F.); angeladinoia@libero.it (A.D.N.); leonardodng1994@gmail.com (L.D.); ad.inchingolo@libero.it (A.D.I.); 2 Implant Dentistry College of Medicine and Dentistry, Birmingham B4 6BN, UK; andrea.palermo2004@libero.it

**Keywords:** malocclusions, orthognathic surgery, relapse, class III skeletal, prognathic mandible, recurrence

## Abstract

Background: This review aimed to analyze the relapse in orthognathic surgery. Methods: PubMed, Scopus, and Web of Science databases were used to find papers that matched our topic dating from 1 January 2012 up to November 2022. Inclusion criteria were (1) human studies, (2) open access studies, (3) studies concerning the correlation between orthognathic surgery and relapse. Exclusion criteria were: (1) in vitro or animal studies, (2) off-topic studies, (3) reviews, (4) other languages than English. Results: A total of 482 results were obtained resulting in 323 publications after duplicate removal (158). After screening and eligibility phases 247 records were excluded: 47 reviews, 5 in animals, 35 in vitro, 180 off-topic. The authors successfully retrieved the remaining 78 papers and evaluated their eligibility. A total of 14 studies from these were ultimately included in the review. Conclusion: Using cephalometric examinations and digital study models, these studies reveal that the relapse after orthognathic surgery is an event that occurs in most of the cases. The limitation of our research is that most of the studies are retrospective and use small sample sizes. A future research goal should be to conduct long-term clinical trials with larger numbers of samples.

## 1. Introduction

Nowadays, severe maxillofacial deformities and asymmetries, with occlusal alterations, are among the main conditions requiring correction by orthognathic surgery [1,2,3,4], an extensive orthodontic and maxillofacial surgical procedure [5,6,7]. Maxillofacial deformities can result from disease, trauma, or genetic predispositions [8]. These deformities cause differences in the size of jaw bones and in the relationship between maxilla and mandible [9,10,11,12]. Common skeletal malocclusion that require orthognathic surgery are class three malocclusions that can be classified into (1) mandibular prognathism, (2) maxillary retrognathism, or (3) both conditions [13,14]. Class III malocclusions are the most difficult maxillofacial deformities to correct due to unfavorable mandibular skeletal growth. This issue affects 7.04 percent of the population overall, and many people have surgery to treat it [15,16].

**Figure 1:**
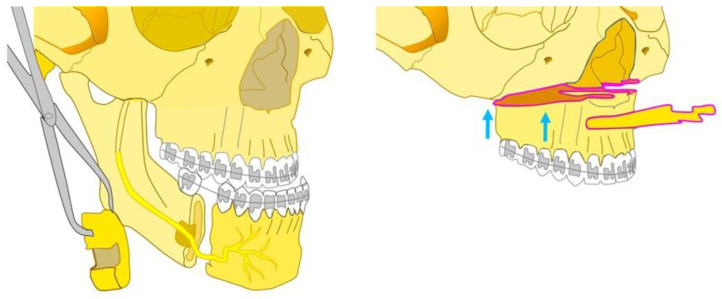
Orthognathic Surgery. Correction of a Class III malocclusion.

A condition often characterized by skeletal class III malocclusion is lip and palate (CL/P) cleft. The most prevalent congenital anomaly of the face is the CL/P, and 20–40% of those affected go on to have a skeletal class III malocclusion due to acquired midface hypoplasia, requiring surgery to be corrected [17,18,19,20]. The affected people usually exhibit maxillary–mandibular skeletal discrepancies, teeth malocclusion, and jaw growth disturbances [21,22]. Although compromised aesthetics is often the patient’s main concern, these malocclusions cause functional problems (due to anterior and posterior cross bites), reduced masticatory performance, problems with breathing and speech, and mild to severe aesthetic impairments, as well as facial deformities, vertical and transversal deficiencies, and lip and nose deformities [23,24,25]. The face is frequently somewhat undeveloped in the inferior region, which typically results in a sunken aspect of the face that destroys facial harmony and has an adverse psychological impact on the patient [26,27]. Due to the appearance of the profile and dental malocclusion, psychological distress leads to shyness and low self-esteem, ultimately negatively affecting the subject’s work and social life [26,28]. The protrusion of the chin and lower lip are indicative of mandibular prognathism, which is most frequently corrected through orthognathic surgery utilizing sagittal split ramus osteotomy (BSSRO) or vertical ramus osteotomy (intraoral: IVRO, extraoral: IVRO) [26,29]. According to the most recent standards of facial aesthetics, one of the most crucial parts of the face is the chin [30,31]. Patients undergo orthognathic surgery to improve masticatory function but mainly for facial aesthetics, to conform to current aesthetic preferences [26,32]. When the surgical treatment is planned, it is important to understand the amount of maxillary advancement required to provide stable, good-looking, and functional results throughout time [33,34]. Whenever the horizontal mismatch exceeds the acceptable threshold for single jaw repair, bimaxillary treatment is performed by compensating for maxillary advancement and mandibular retrusion [35,36,37] (Figure 1).

However, when it moves forward with a counterclockwise spin, the mandible has a tendency to collapse backward. Orthognathic surgery is often necessary in addition to orthodontic treatment for the resolution of an important bimaxillary dentoalveolar alteration [38]. Le Fort 1 maxillary osteotomy (LF1) and anterior subapical mandibular osteotomy (ASO) are surgical procedures to limit the drawbacks of orthodontic therapy alone [39,40].

**Figure 2:**
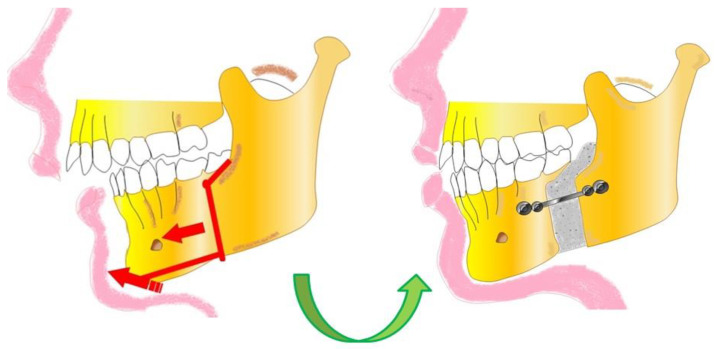
Orthognathic Surgery. Correction of a severe skeletal Class II malocclusion.

Severe skeletal Class II malocclusions often need surgical treatment to obtain an optimum balance between skeletal structures and the soft tissue. Surgical orthodontic intervention is an effective option for treatment of adults with severe maxillary protrusion (Figure 2).

In these cases although ASO is very useful, it is still very limited in the treatment of severe maxillary protrusion [41,42]. Many studies consider individuals with various dentofacial deformities by analyzing the ASO together with other osteotomies [43]. Mandibular surgery single (advancement or retraction) with bilateral split ramus osteotomy (BSSRO) is a technique that is frequently used for the management of skeletal deformities with a mandibular component [44,45]. Stability of the results over time is a key indicator of the effectiveness of these techniques [40,46]. Relapse is defined as any type of loss of skeletal or dental corrections achieved during treatment [47]. The kind and extent of the motions of the maxilla, which are complicated in people with CL/P, determine the stability of orthognathic surgery [48,49]. Postoperative complications consist, for example, in nerve injury, infections, hemorrhage, and in skeletal relapse [50,51]. The most significant problem is postoperative skeletal relapse in patients undergoing orthognathic surgery [26], although the relapse rate has decreased with the post-treatment outcomes after the introduction of rigid internal fixation [52,53]. Relapse is a process that is ongoing and is caused by a variety of short- and long-term variables [54]. Condylar morphological abnormalities, muscular tension brought on by excessive surgical motion, and improper placement of the condyles in the glenoid fossa during surgery are the primary causes of short-term relapse [55]. Long-term relapse is instead attributed to progressive changes, resorption and adaptation in the condyles, and continued skeletal growth [56,57]. This study analyzes the relapse related to the various orthognathic surgery procedures, in which postoperative phase they occur, and their percentage [58,59].

## 2. Materials and Methods

### 2.1. Search Processing

The PRISMA guidelines were followed in doing this systematic review [60] and it has been registered on PROSPERO under ID 442578. Studies on this subject from 1 January 2012 through 22 November 2022 were searched for in PubMed, Scopus, and Web of Science with an English language constraint. This research’s major emphasis is the utilization of orthognathic surgery and the potential for relapse, hence a search strategy was developed using a mix of phrases that matched those objectives (“Relapse” AND “Orthognathic Surgery”).

Despite the value of any inclusion of NNT (number needed to treat), 95% CIs (confidence intervals), risk analysis, and NNH (number needed to harm) in the study, the main topic of our review is the qualitative examination of the body of literature on orthognathic surgery and recurrence. As a result, our main goal was to analyze qualitatively and not statistically the relapse associated with the various orthognathic surgery techniques, the postoperative phase in which the relapse occurred, and their proportion.

### 2.2. Inclusion and Exclusion Criteria

The inclusion criteria are listed below: (1) human studies, (2) full-text-available studies, (3) studies concerning the relapse after orthognathic surgery. The following were the exclusion requirements: in vitro or animal studies, off-topic research, book chapters, reviews, and non-English language studies were the first four categories.

## 3. Results

**Figure 3:**
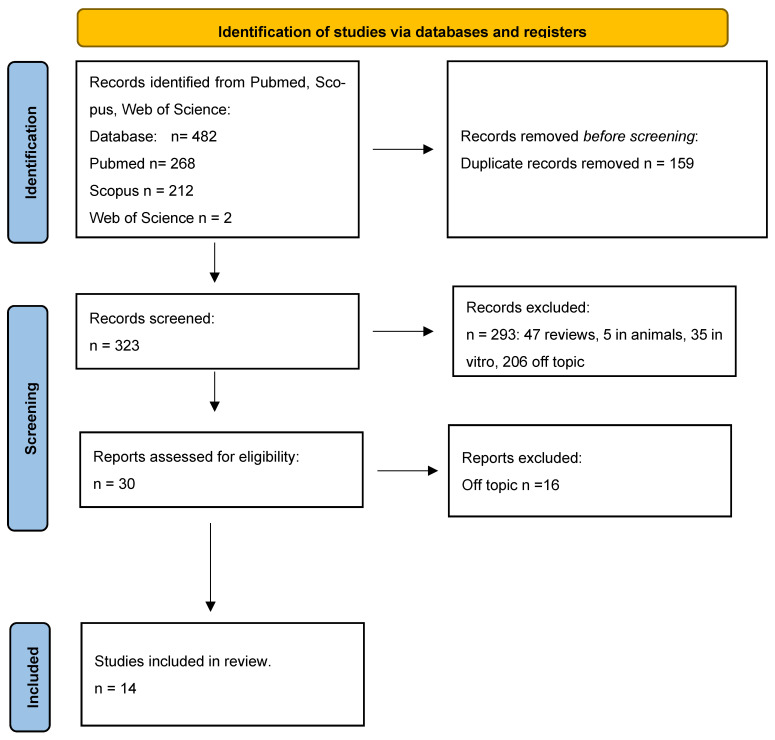
PRISMA flow chart.

The following database yielded a total of 482 publications, including PubMed (268), Scopus (212), and Web of Science (2). After 158 duplicates were removed, 323 articles remained. A total of 293 records were excluded by analysis of the title or abstract: 47 reviews, 5 animals, 35 vitro, 206 off-topic. The reports assessed for eligibility were 30. From these, 16 articles were off topic, so they were excluded, and finally, the studies included in this review are 14 (Figure 3) (Table 1). In summary, from 482 initial articles 14 articles were used for this review.

## 4. Discussion

Relapse is a potential risk after orthognathic surgery [68]. The incidence of relapse after orthognathic surgery has been the subject of extensive investigation in recent years and it is a continuous process that needs to be assessed both now and in the future [47]. Compared to the general population, the risk of relapse is greater in CL/P patients due to more risk factors [63]. The association between CL/P and a higher likelihood of recidivism is well acknowledged, even though additional causes are not fully understood [63]. In fact, in a study by da Silva et al., even though the overjet values previous to surgery and the degree of maxillary advancement were identical in the groups with and without cleft, it was found that patients who had CL/P had an average relapse of 1248 cm more than patients who did not have CL/P [63].

The first few days following surgery are quite challenging for the patients [61]. Following the orthognathic surgery treatment, the postoperative healing period might take weeks or months [35]. The detection of relapse and its complex effect can be minimized by identifying their causes [66].

A study by Sahoo N. K. et al. [47] was carried out by evaluating the registration of the treatments of 46 patients undergoing mandibular orthognathic surgery, either advancement (group 1, 26 subjects) or mandibular retrusion (group 2, 20 subjects) [47]. It was based on the analysis of some parameters of postoperative relapse using cephalometry [47]. At T0 (one week before surgery), T1 (one week following surgery), T2 (one year following surgery), and T3 (five years following surgery), lateral cephalograms were plotted [47]. Relapse control was carried out in the horizontal, vertical, and angular parameters studied in group 1. Through the study of these parameters it emerged that there was a rapid and significant relapse (from T1 to T2) which lasted until the long-term evaluation (from T2 to T3) (*p* value\0.0001) [47]. Regarding the short-term and long-term relapse assessment studied horizontally, vertically, and angularly in group 2, the values show a significant short-term (T1 to T2) relapse that significantly continued until the long-term evaluation (T2 to T3) (*p* value\0.0001). Mean linear vertical relapse (T1–T3 and T1–T3) was higher in group 2 than in group 1 in all parameters (except for Pog and overbite at T1–T2) (*p* value\0.005 for all) [47]. Mean angular relapse in all (T1–T2 and T1–T3) was higher in all parameters (except ramus inclination at T1–T2) in group 2 compared to group 1 (*p*-value\0.005 for all) [47]. Relapse was correlated with gender, age, surgical displacement performed during surgery, and mandibular angle change occurring intraoperatively [47]. Regarding the correlation of relapse with gender, and age, the relapse at the time T1–T2 and T1–T3 did not show a statistically significant positive correlation with gender or age in either group (*p* value 0.005 for all) [47].

### 4.1. Le Fort 1 Osteotomy

It was demonstrated that the bone graft that is inserted into the gap left by the Le Fort 1 osteotomy protected against jaw relapse [63]. In fact, comparing the data in the two sample groups, it was shown that patients receiving bone transplants had an average 1.723 mm less relapse [63]. Bone from the patient’s jaw can be used for this autologous graft [63]. The patients all had a skeletal class three malocclusion and required maxillary advancement surgery with the Le Fort 1 technique (single jaw advancement), or Le Fort 1 with BSSO (bilateral jaw surgery) [69]. Prior to surgery, the patients who had undergone bimaxillary surgery had greater mean negative overjet values [35]. However, there was no significant difference in the amount of mean maxillary advancement between the two patient groups (those who underwent bimaxillary surgery and those who underwent the Le Fort 1 procedure) [35].

Another study discussed the relapse following Le Fort 1 osteotomy for maxillary advancement in individuals with oral CL/P, as well as maxillary hypoplasia [63]. In fact, the most common surgical treatment for maxillary retrusion is the Le Fort 1 maxillary osteotomy even if this line of treatment is unstable due to the kind and the width of the maxillary movements [63]. In that study, researchers examined whether patients with oral clefts who underwent maxillary advancement surgery (Le Fort 1 maxillary osteotomy) tend to have their teeth and bones shift back to their original positions [63]. Using the program Dolphin 3D, the lateral cephalograms were digitally analyzed evaluating vertical and horizontal measures, at three different times: T1 (before the orthognathic surgery), T2 (immediately after the orthognathic surgery), and T3 (six months/one year after the orthognathic surgery) [63]. In addition to skeletal stability, the stability of the teeth is a critical component for the treatment’s success in the evaluation of relapse after orthodontic surgery [63]. The study revealed that following osteotomy Le Fort 1, there was a maxillary relapse in the vertical direction (100%) but not in the horizontal direction, and dental measures were taken throughout the study period [63]. There was no horizontal relapse at 6 months or 1 year following maxillary advancement surgery (Le Fort 1), and there was no relapse of the overbite, overjet, or tooth midline deviation at 2 years [63]. The relationship between the right and left premolars was good and steady [63]. In a different investigation, the authors assessed the anticipated 10–50% maxillary horizontal relapse following Le Fort 1 progress [35]. They hypothesized that it is proportional to maxillary advancement [35].

The Le Fort 1 osteotomy and the segmental Le Fort 1 osteotomy are two surgical methods frequently used to expand the upper jaw [35]. Both of these surgical methods cause relapse after surgery, as has been seen with all previous surgical methods [35]. Because ligaments and soft tissue have a tendency to return to their pre-injury state, the great majority of relapses take place as a result of this [35]. While performing osteotomy Le Fort 1, the upper side of the jaw bone does not contain any relapse-inducing structures [61]. With the Le Fort 1 segmental osteotomy it is possible to have expansion in any direction [61]. The intercanine breadth and the related anterior skeletal width (width of the piriform aperture) were thus measured by certain authors [61].

By conducting a study with a control group (Le Fort 1 osteotomy) and an experimental group (segmental Le Fort 1 osteotomy), skeletal and dental relapse after Le Fort 1 osteotomy was assessed in adult patients with class III malocclusions who needed maxillary expansion [63]. Unfortunately, surgical plates placed near the pyriform aperture caused image artifacts and, as a result, the intercanine width could not be accurately measured [63]. Easily accessible autologous bone grafting from the mandible or maxilla into the gap left by the Le Fort 1 osteotomy is recommended because it appears to stop maxillary relapse [63]. In conclusion, good postoperative stability is achieved with Le Fort 1 segmental osteotomy surgery, with a skeletal relapse rate of 26% at 12 months [63]. On the other hand, augmentation of expansion, which can be achieved by various means (the use of resorbable plates in the pacemaking region, bone grafting in the expansion area, or placement of a palatal arch), could serve to prevent relapse [63].

### 4.2. BSSO (Bilateral Sagittal Split Osteotomy)

The surgical procedure of choice for patients with severe discrepancies, i.e., for those that have an advancement of more than 6.7 millimeters that needs to be corrected, is bimaxillary surgery. One of the results a previous study achieved was the protection against jaw relapse provided by a bone graft that was placed into the gap created by the Le Fort 1 osteotomy [63].

In a different study, the authors compared the long-term skeletal stability of two groups of patients having surgery on the mandibular sagittal split ramus in relation to the use of miniplates: resorbable meshes (hydroxyapatite/poly-l-lactide) and titanium miniplates [70]. When compared to the titanium-fixation group, the HA/PLLA showed greater long-term skeletal stability concerning the location of the mandible [70]. A study on the adaptability of sagittal curved osteotomy as an alternative to the traditional approach in patients with retrognathism explored the potential of genioplasty. Twenty-four patients were randomly divided in two groups: group 1, patients in who sagittal curving osteotomy was performed, and group 2, in who conventional osteotomy was performed [71]. Relapses in both hard and soft tissues were studied between two groups [71]. Following genioplasty, sagittal curved osteotomy may assist to reduce relapse [71].

### 4.3. Combined Maxillomandibular Approach

By using a modified orthodontic and surgical approach, severe class III skeletal deformity and malocclusion could be successfully treated, and facial balance and symmetry improved [66]. Orthognathic surgery for mandibular prognathism, which reduces the gap between the jaws, enables proper occlusion, enhances masticatory function, and improves the appearance of the smile [66]. BSSRO and IVRO are the most widely used methods [66]. Therefore, the stability of the mandible after surgery is fundamental [66]. The degree of retreat, the surgical approach, the intersegmental fixing technique, and the postoperative condylar position all influence postoperative mandibular stability [26]. In a study it was found that in the immediate postoperative phase (T21), group A experienced an immediate retrusion of 15.55 mm,, which is significantly greater than group B’s (10.97 mm) [26]. At the final follow-up (T32), group A demonstrated a significant reduction of 4.07 mm, whereas group B demonstrated a significant posterior derivation of 1.23 mm [26]. The cut-off point of the retraction that would cause a clinical relapse of 2 mm was discovered to be 14.1 mm in the analysis of the ROC [26].

In a study by Tai et al. the correction of bimaxillary protrusions was carried out by increasing the inter-incisive angle by 21.1° [43]. A medium reduction in L1-MP occurred following the straightening of the anterior subapical osteotomy segments [43]. The majority of patients (96.7%) had L1-MP relapses two years after the intervention; however, the mean effect size of 2.9° was probably not clinically significant [43]. For assessing 2-year stability, no particular risk indicators for relapse could be found [43].

After orthognathic surgery, the necessity for TMJ surgery was assessed in a retrospective cohort analysis. Individuals with internal derangement only showed significant occlusal abnormalities in one patient, as opposed to individuals with bicondylar resorption, in whom the skeletal relapse remained a cause for concern [65].

Last but not least, our study discovered a case report, the first instance in the literature, of a patient with radiographically well-documented myotonic dystrophy who received a combined orthodontic and orthognathic surgical therapy and had a long-term follow-up. This 17-year-old boy had a relapse, not related to surgical therapy, but due to his skeletal and muscular issues: long, tapered face, pronounced open bite, and type 1 MD. Because of the open bite and weak musculature, there were long-term stability problems [67].

Faharadyan and colleagues’ research disproved any link between maxillary progress and relapse [35]. As a result, overcorrection should be taken into account [35]. Their findings show that there is a positive correlation between maxillary advancement and horizontal relapse as well as maxillary relapse in both the horizontal and rotational directions [35].

Modified intraoral osteotomy was designed to decrease the stress on the condyles by reducing the risk of relapse after condylar resorption [62].

In a different study, adult patients with class III skeletal malocclusion who required jaw expansion surgery had their skeletal and dental widths measured quantitatively using CBCT after Le Fort 1 segmental osteotomy [61].

According to studies, 63–73% of class III malocclusions are skeletal in nature [24]. A concave facial profile is caused by such skeletal abnormalities, which are brought on by an imbalance in the mandibular and maxillary growth in people from a lower social class [24].

## 5. Conclusions

In summary, it is evident from a comparison of the different papers included in the eligibility that recurrence is a constant in the post-surgical course after an orthodontic surgery. Recurrences have been observed most frequently within six months from the operation and, in any event, within a year. The use of bone grafts in the bone gap established in Le Fort 1 or the use of absorbable plates rather than titanium plates is an example of factors that lower the risk of relapse. As opposed to that, it is easier to have a relapse when it is necessary to make large mandibular advances, and it makes no difference whether a third-grade patient has a CL/P or not. The limitation of our research is that most of the studies were retrospective; therefore, our study also has the limitations linked to these studies, which are that study events already occurred and the fact that only studies with small sample sizes were available. For the future, it is hoped to have RCT studies with large sample sizes. Future research goals should be to conduct long-term clinical trials with larger sample sizes.

## Figures and Tables

**Table 1:** Characteristics of the in vivo studies included in the qualitative analysis.

Authors and Year	Type	Aim	Materials and Methods	Results
Kim et al., 2018 [61]	Randomized clinical study	In adult patients with skeletal class III malocclusion who need maxillary expansion, to use cone-beam computed tomography (CBCT) to assess the stability of the skeletal and dental widths after segmental Le Fort 1 osteotomy.	Le Fort 1 osteotomies (control group) and segmental Le Fort 1 osteotomies (experimental group) were performed on 25 and 36 patients with skeletal class III malocclusion, respectively. The skeletal and dental widths were measured on CBCT pictures (T1, T2, and T3) before, after, and at the end of the course of therapy. It was shown that the degree of relapse in the experimental group and the extent of surgery were correlated.	In the experimental group, the amount of segmental Le Fort 1 skeletal expansion was inversely correlated with the degree of postoperative skeletal relapse.
Fahradyan et al., 2018 [35]	Prospective study	Examine the relationship between the degree of maxillary advancement and relapse.	Between 2008 and 2015, bimaxillary surgery and either a Le Fort 1 or a Le Fort 1 with mandibular setback were performed on individuals with class III skeletal malocclusions.	The horizontal relapse was 1.8 mm and the mean maxillary advancement was 6.3 mm for a relapse of 28.6%.
Sahoo. et al., 2020 [47]	Study in vivo	Think about relapse in the long term compared to the short term.	46 patients who underwent mandibular orthognathic surgery had their medical records split into two categories, surgery for mandibular advancement and surgery for mandibular setback.	The amount of surgical movement and the intraoperative change in mandibular plane angle were substantially linked with relapse in both groups (*p* values for each were 0.05).
da Costa Senior et al., 2021 [62]	Study in vivo	This study’s objective was to assess how well the surgical technique addresses condilar relapse.	7 patients underwent bilateral sagittal split osteotomies, and 2 additional Le fort 1 osteotomies and TMJ surgeries were performed in 2 cases.	Patients who require additional orthognathic surgery and those who experience malocclusion after condylar resorption may find relief with the modified C-osteotomy.
da Silva et al., 2018 [63]	Retrospective study	To evaluate and find relapse after orthognathic surgery for maxillary advancement (Le Fort 1 maxillary osteotomy in oral cleft patients two years later); to analyze digital cephalograms and three-dimensional dental models.	Dental casts and lateral cephalograms were performed on 17 people. The digital cephalometric tracings were assessed in T1 (before surgery), T2 (immediately after surgery), and T3–6 months to 1 year after surgery. The dental casts are displayed in F1, F2, and F3.	While the other parameters under investigation were unaffected, cephalometry revealed a relapse in the vertical movement following maxillary advancement utilizing orthognathic surgery.
Al-Delayme et al, 2018 [64]	Prospective comparative clinical trial	Assess the postoperative stability of the double-jaw surgical treatment of skeletal class III deformities, and compare the two distinct mandibular surgical procedures.	12 patients with skeletal class III malocclusions were included in this study. The patients underwent BSSO or IVRO in addition to a Le Fort 1 osteotomy for double-jaw surgery. Prior to T0, immediately after the procedure (T1), and one year later, lateral cephalograms were performed.	The average mandibular setback and maxillary advancement in the BSSO group were respectively 6.22 mm at B point and 2.93 mm at point A, with relapse rates of 24.9 and 26.6.
Politis et al., 2018 [65]	Retrospective cohort study	After orthognathic surgery, assess the need for TMJ surgery.	630 patients underwent Le Fort 1 osteotomies or sagittal split osteotomies between January 2013 and December 2016.	Individuals with internal derangement only showed severe occlusal anomalies in one case, unlike those with bilateral condylar resorption, where the skeletal relapse persisted as a problem.
Peleg et al., 2022 [66]	Retrospective cohort study	During orthognathic surgery, look into mandibular operations, paying close attention to the two most common procedures between January 2010 and December 2019: IVRO and SSO.	There were 144 patients altogether. IVRO:SSO procedures were 118:26 in number.	Overall, there were 53 problems/issues following surgery, such as skeletal relapse, temporomandibular joint dysfunction, etc.
Antonarakis., et al., 2019 [67]	Case report	To provide the first case of combined orthodontic and orthognathic surgical therapy for a patient with MD who has had a lengthy, well-documented follow-up using radiography.	Orthognathic and orthodontic surgery were performed on a 17-year-old male patient who had a significant open bite, a long, tapering face, and MD type 1.	Long-term stability issues in a patient with MD who underwent orthognathic surgery and orthodontic treatment to close his anterior open bite are discussed.
Tai Wayne et al., 2022 [43]	Retrospective study	To assess the stability and negative consequences of mandibular anterior subapical osteotomy (ASO) as a therapy for bimaxillary dentoalveolar protrusion.	Between 2008 and 2017, 120 individuals who underwent orthognathic surgery at a single hospital were included. Serial lateral cephalogram traces were taken prior to surgery (T1), six weeks after surgery (T2), and two years following surgery in order to evaluate relapse.	L1-MP increased on average by 12, 7°. At 2 years following surgery, 96.7% of patients had a mean L1-MP relapse of 2.9°. There was no clear factor that enhanced the chance of relapse and the degree of surgical repositioning was only sporadically connected with that of relapse, etc.
Peleg et al., 2022 [66]	Retrospective cohort study	During orthognathic surgery, look into mandibular operations, paying attention to the two most common procedures between January 2010 and December 2019: IVRO and SSO.	There were 144 patients altogether. IVRO:SSO procedures were 118:26 in number.	Overall, there were 53 problems/issues following surgery, such as skeletal relapse, temporomandibular joint dysfunction, etc.

## Data Availability

Not applicable.

## Abbreviations

(CL/P) cleft lip and palate, (LF1) Le Fort 1, (ASO) anterior subapical mandibular osteotomy, (TC) third class malocclusion, (BSSRO) bilateral sagittal split ramus osteotomy, IVRO (intraoral vertical ramus osteotomy), EVRO (extraoral vertical ramus osteotomy), HA/PLLA (hydroxyapatite/poly-l-lactic acid), MD (myotonic dystrophy).
